# Acoustic-Frequency Vibratory Stimulation Regulates the Balance between Osteogenesis and Adipogenesis of Human Bone Marrow-Derived Mesenchymal Stem Cells

**DOI:** 10.1155/2015/540731

**Published:** 2015-02-08

**Authors:** Xi Chen, Fan He, Dong-Yan Zhong, Zong-Ping Luo

**Affiliations:** ^1^Orthopaedic Institute, Soochow University, Department of Orthopaedics, the 1st Affiliated Hospital of Soochow University, Suzhou, Jiangsu 215007, China; ^2^School of Biology and Basic Medical Sciences, Medical College, Soochow University, Suzhou, Jiangsu 215123, China

## Abstract

Osteoporosis can be associated with the disordered balance between osteogenesis and adipogenesis of bone marrow-derived mesenchymal stem cells (BM-MSCs). Although low-frequency mechanical vibration has been demonstrated to promote osteogenesis, little is known about the influence of acoustic-frequency vibratory stimulation (AFVS). BM-MSCs were subjected to AFVS at frequencies of 0, 30, 400, and 800 Hz and induced toward osteogenic or adipogenic-specific lineage. Extracellular matrix mineralization was determined by Alizarin Red S staining and lipid accumulation was assessed by Oil Red O staining. Transcript levels of osteogenic and adipogenic marker genes were evaluated by real-time reverse transcription-polymerase chain reaction. Cell proliferation of BM-MSCs was promoted following exposure to AFVS at 800 Hz. Vibration at 800 Hz induced the highest level of calcium deposition and significantly increased mRNA expression of *COL1A1*, *ALP*, *RUNX2*, and *SPP1*. The 800 Hz group downregulated lipid accumulation and levels of adipogenic genes, including *FABP4*, *CEBPA*, *PPARG*, and *LEP*, while vibration at 30 Hz supported adipogenesis. BM-MSCs showed a frequency-dependent response to acoustic vibration. AFVS at 800 Hz was the most favorable for osteogenic differentiation and simultaneously suppressed adipogenesis. Thus, acoustic vibration could potentially become a novel means to prevent and treat osteoporosis.

## 1. Introduction

Osteoporosis is a systemic disease characterized by low bone mass and deterioration of the bone microarchitecture, resulting in increased bone fragility and susceptibility to fracture. Worldwide, 9 million people suffer from osteoporosis-related fractures every year [1] and more importantly, osteoporosis-related fractures are a major cause of morbidity and mortality. For example, in the United States, more than 30,000 deaths within half a year are imputed to hip fractures [2]. Current osteoporosis treatments predominantly use bone-antiresorptive drugs that are associated with several side effects without fully restoring the balance between bone resorption and formation [3, 4].

The disorders of bone remodeling are accompanied by an increase in marrow fat and a decline in bone mass [5]. The balance between osteogenesis and adipogenesis of bone marrow-derived mesenchymal stem cells (BM-MSCs), as the common progenitors of osteoblasts and adipocytes [6], plays an important role in maintaining bone homeostasis. Therefore, the shift of BM-MSC differentiation into the adipocyte lineage may result in inadequate bone formation and excessive fat production [7]. The differentiation of BM-MSCs is regulated by transcription factors, which affect the lineage fate determination. More specifically, peroxisome proliferator-activated receptor-*γ* (PPAR-*γ*) promotes adipogenesis while inhibiting osteogenesis, whereas runt-related transcription factor 2 (Runx2) enhances osteogenesis instead of adipogenesis. PPAR-*γ* can suppress the activity of Runx2 and thus inhibits expression of osteogenesis-related genes [8].

The growing number of evidence has demonstrated the role of both biochemical and biomechanical signals in regulating MSC lineage-specific differentiation, where mechanical stimuli affect the metabolism of bone cells and their precursors. Low-frequency vibration can enhance bone remodeling, prevent bone loss, and improve bone healing in both animals and humans [9]. Furthermore, mechanical stimulation can direct the lineage commitment of MSCs by biasing cell fate in favor of osteogenesis, suggesting that MSCs may sense the vibratory frequencies [10–12]. Luu et al. reported that a daily exposure to low magnitude mechanical signals facilitated MSC proliferation and osteogenic differentiation [13]. Presently, little is known about the simultaneous regulation of mechanical vibration, especially at acoustic frequencies, on osteogenesis and adipogenesis of BM-MSCs, which significantly limits our understanding of the role of the mechanical stimuli on this critical osteoporotic mechanism.

In this study, we investigated the role of acoustic-frequency vibratory stimulation (AFVS) at 30, 400, and 800 Hz, which generated the same vibration amplitude of the cell culture plates, in regulating proliferation, osteogenesis, and adipogenesis of BM-MSCs. Expression of adipogenic and osteogenic marker genes, their functional capacity to form mineralized extracellular matrix (ECM), and lipid accumulation in the differentiated cells were examined. We hypothesized that the responses of BM-MSCs to acoustic vibration could highly depend on the frequency and that the higher frequency vibration could determine the lineage fate of MSCs.

## 2. Materials and Methods

### 2.1. Cell Culture of BM-MSCs

Human BM-MSCs (American Type Culture Collection, Manassas, VA, USA) were cultured in 175 cm^2^ cell culture flasks (Corning, Tewksbury, MA, USA) in modified Eagle's medium alpha supplemented with 10% fetal bovine serum (FBS), 100 U/mL penicillin, and 100 *μ*g/mL streptomycin (GIBCO, Carlsbad, CA, USA) at 37°C in a humidified 5% CO_2_ atmosphere. Upon reaching 70% confluence, cells were dissociated by 0.25% trypsin-EDTA (Invitrogen Carlsbad, CA, USA) and reseeded into 96-well and 12-well plates for the next stage of the experiments.

### 2.2. Acoustic Frequency Loading Platform

A vibration loading of cell cultures was developed to stimulate BM-MSCs with different vibrations. 12-well plates cultured with BM-MSCs were mounted onto the platform of a vibration shaker (U8556001, 3B Scientific, Hamburg, Germany), which was excited by one channel of functional generator (ATF20B, ATTEN, Shenzheng, China) providing the amplitude, waveform, and frequency of the vibration. When the platform was placed in the cell culture incubator, BM-MSCs cultures received vertical sinusoid stimuli (magnitude of 0.3 g, frequencies of 0, 30, 400, and 800 Hz) for 30 min per day [14, 15].

### 2.3. Cell Viability Stain and Cell Proliferation Assay

1000 cells/well of BM-MSCs were seeded in a 96-well flat-bottomed plate at 37°C in 5% CO_2_. The cells were treated with acoustic vibratory stimulation for 30 min each day at the frequency of 0, 30, 400, or 800 Hz, in hexaplicate wells. After 7 days, cell viability was assessed by fluorescein diacetate (FDA; Sigma-Aldrich, St Louis, MO, USA) staining [16]. Cells were incubated in 5 *μ*g/mL FDA solution at 37°C for 10 min and fluorescence images were captured with an Olympus IX51 microscope (Olympus Corporation, Tokyo, Japan). Cell proliferation was evaluated using the Cell Counting Kit-8 (CCK-8; Beyotime Institute of Biotechnology, Haimen, China). Briefly, 10 *μ*L of the CCK-8 solution was added to each well and the cells were incubated at 37°C for 1 h. Absorbance was determined at 450 nm using a microplate spectrophotometer (BioTek, Winooski, VT, USA).

### 2.4. Osteogenic Differentiation of BM-MSCs and Alizarin Red S Staining

To induce BM-MSCs into osteoblasts, cells were cultured in osteogenic differentiation medium (DMEM (low glucose) supplemented with 10% FBS, 100 U/mL penicillin, 100 *μ*g/mL streptomycin, 2 × 10^−4^ M L-ascorbic acid, 10^−7^ M dexamethasone, and 10^−2^ M *β*-glycerophosphate disodium salt hydrate (Sigma-Aldrich)) for 14 days [17, 18]. The differentiation medium was changed every 3 days.

Calcium deposition in the extracellular matrix was determined by Alizarin Red S staining after 7 and 14 days of osteogenic differentiation. Cells were fixed in 4% polyoxymethylene (Sigma-Aldrich) and then incubated in 1% Alizarin Red S solution (pH = 4.3; Sigma-Aldrich). Images of calcium deposition were captured with an Olympus IX51 microscope. To quantify the calcium deposition, 200 *μ*L/well of 1% hydrochloric acid (Sigma-Aldrich) was added and absorbance was measured at 420 nm using a microplate spectrophotometer (BioTek).

### 2.5. Adipogenic Differentiation of BM-MSCs and Oil Red O Staining

To induce BM-MSCs into adipocytes, cells were cultured in adipogenic differentiation medium (DMEM [high glucose] supplemented with 10% FBS, 100 U/mL penicillin, 100 *μ*g/mL streptomycin, 10^−6^ M dexamethasone, 5 × 10^−4^ M 3-isobutyl-1-methylxanthine, 10 mg/L insulin, and 10^−4^ M indomethacin (Sigma-Aldrich)) for 21 days [17]. The differentiation medium was changed every 3 days.

The differentiated BM-MSCs were stained with Oil Red O after 14 and 21 days of adipogenic differentiation. The induced adipocytes were fixed with 10% buffered formaldehyde (Sigma-Aldrich) and then incubated in Oil Red O solution (Sigma-Aldrich) for 30 min at room temperature. Images of adipocytes were captured with an Olympus IX51 microscope. To quantify the induced adipocytes, 200 *μ*L/well of isopropanol was added and absorbance was measured at 510 nm using a microplate spectrophotometer (BioTek).

### 2.6. Total RNA Extraction and Real-Time Reverse Transcription-Polymerase Chain Reaction (Real-Time RT-PCR)

Total RNA was extracted (from four groups of cells per experimental condition) using TRIzol reagent (Sigma-Aldrich) and 1 *μ*g of total RNA was reverse-transcribed using the RevertAid First Strand cDNA Synthesis Kit (Thermo Fisher Scientific). To quantify mRNA expression, an amount of cDNA equivalent to 20 ng of total RNA was amplified by real-time PCR using the iTap Universal SYBR Green Supermix kit (Bio-Rad, Hercules, CA, USA). Transcript levels of osteogenic marker genes (*ALP*,* COL1A1*,* SPP1/OPN*, and* RUNX2*), adipogenic marker genes (*FABP4/αP2*,* CEBPA/C/EBP-α*,* LEP/Leptin*, and* PPARG2/PPAR-γ2*), and* GAPDH* (an internal standard) were evaluated using the primer sequences listed in Table 1. Real-time PCR was performed on a CFX96 Real-Time PCR System (Bio-Rad) following the manufacturer's protocol. Relative transcript levels were calculated as *χ* = 2^−ΔΔCt^, in which ΔΔCt = Δ*E* − Δ*C*, Δ*E* = Ct_exp⁡_ − Ct_GAPDH_, and Δ*C* = Ct_ct1_ − Ct_GAPDH_.

### 2.7. Statistical Analysis

Data from each experiment were expressed as the mean ± standard error (S.E.). Statistic differences between groups were determined by one-way analysis of variance (ANOVA) followed by Student's unpaired *t-*test using the SPSS version 13.0 (SPSS Inc, Chicago, IL, USA). Significance was indicated by a *P* value of <0.05 or <0.01 from Student's unpaired *t*-test.

## 3. Results

### 3.1. The Effect of Acoustic Vibration on Cell Proliferation of BM-MSCs

**Figure 1:**
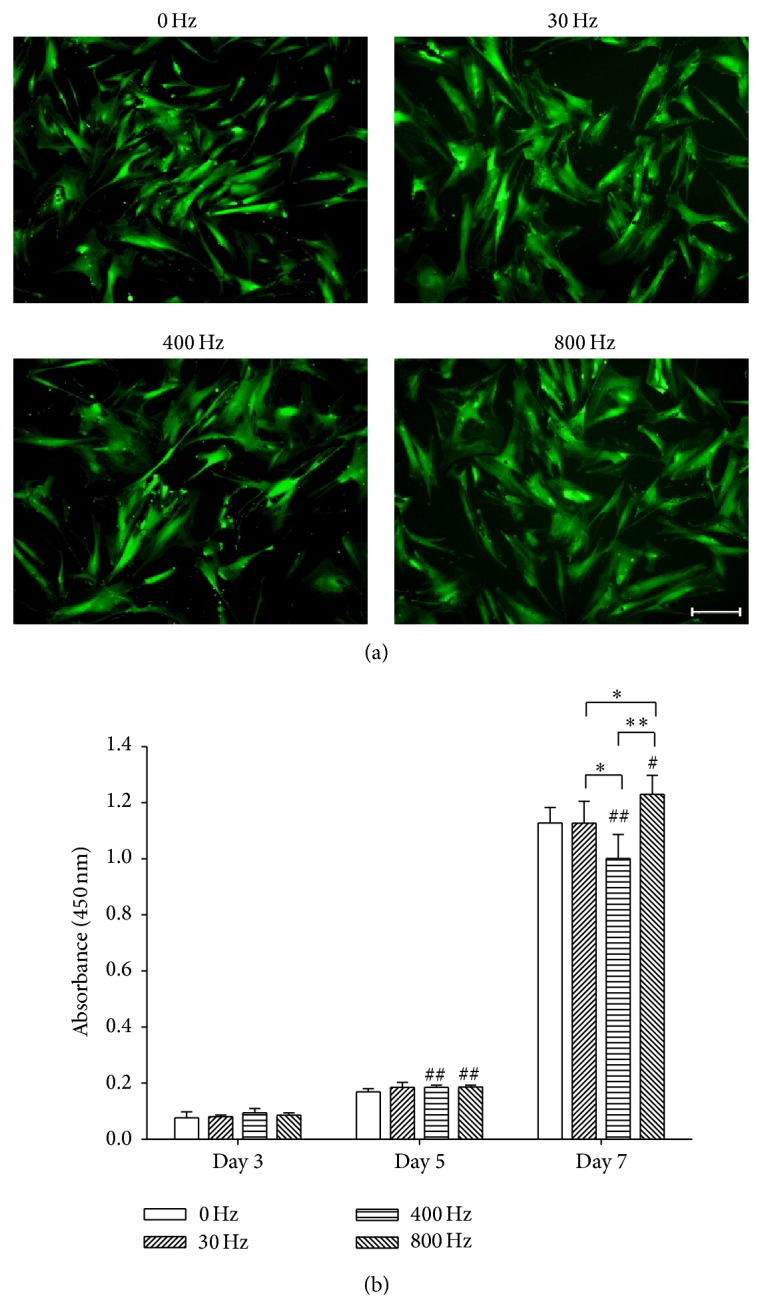
Effects of acoustic-frequency vibratory stimulation (AFVS) on cell viability and proliferation of human bone marrow-derived mesenchymal stem cells (BM-MSCs). (a) Fluorescein diacetate stain showed that BM-MSCs maintained spindle and fibroblast-like cell shape when stimulated with AFVS at the frequencies of 0, 30, 400, and 800 Hz. Scale bar = 200 *μ*m. (b) Cell proliferation of BM-MSCs was improved by AFVS at 800 Hz on day 7. Values are mean ± standard error of six independent experiments (*n* = 6). ^*^ *P* < 0.05, ^**^ *P* < 0.01 in the indicated groups from unpaired *t*-test. ^#^ *P* < 0.05, ^##^ *P* < 0.01 compared with the 0 Hz control group at the same time point from unpaired *t*-test.

BM-MSCs were cultured in growth medium for 7 continuous days and stimulated with AFVS at 0, 30, 400, and 800 Hz. FDA staining showed the cell viability and BM-MSCs treated by AFVS at different frequencies maintained spindle and fibroblast-like cell shape (Figure 1(a)). At the time point of 3, 5, and 7 days, cell proliferation was assessed by CCK-8. On day 5, cell growth was higher by 13.6% in the 400 Hz group and by 10.5% in the 800 Hz group compared with the static group. On day 7, AFVS at 800 Hz induced the highest level of cell proliferation, 9.0% more than the 0 Hz group, 9.1% more than the 30 Hz group, and 22.8% more than the 400 Hz group (Figure 1(b)).

### 3.2. AFVS at 800 Hz Promotes Osteogenic Differentiation in BM-MSCs on Day 14

**Figure 2:**
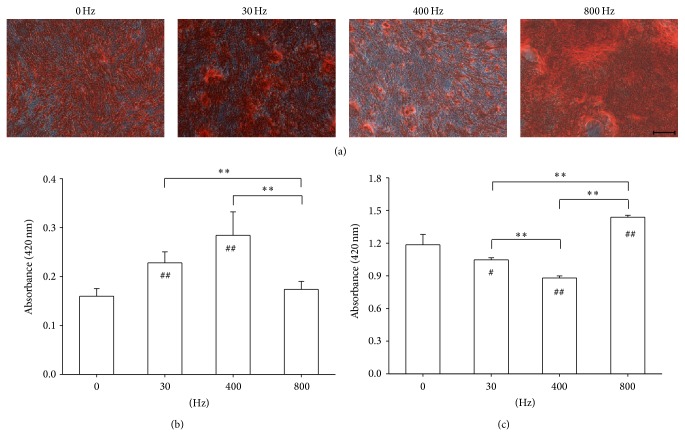
Effects of acoustic-frequency vibratory stimulation (AFVS) on mineralization of the extracellular matrix. Human bone marrow-derived mesenchymal stem cells (BM-MSCs) were cultured in osteogenic differentiation medium for 14 days and stimulated with AFVS at the frequencies of 30 Hz, 400 Hz, and 800 Hz. Cells in static culture condition (0 Hz) served as a control. (a) At the time point of day 14, calcium deposition was assessed by Alizarin Red S staining. Scale bar = 200 *μ*m. The stained mineral layers at the time point of day 7 (b) and 14 (c), respectively, were dissolved in 1% hydrochloric acid and quantified via a spectrophotometer. Values are mean ± standard error of four independent experiments (*n* = 4). ^*^ *P* < 0.05, ^**^ *P* < 0.01 in the indicated groups from unpaired *t*-test. ^#^ *P* < 0.05, ^##^ *P* < 0.01 compared with the 0 Hz control group from unpaired *t*-test.

BM-MSCs were induced toward osteogenesis with the treatment of AFVS at 0, 30, 400, and 800 Hz. Cells in static culture condition (0 Hz) served as a positive control. Mineralization of the ECM, as a typical marker for osteogenic differentiation, was measured by Alizarin Red S staining on day 7 and day 14. AFVS at 800 Hz on day 14 stimulated a denser staining compared to the other three groups (Figure 2(a)). On day 7, AFVS at 30 Hz and 400 Hz increased the level of mineralization by 42.7% and 77.8%, respectively, compared with the static group, whereas significant difference was not detected between the 0 Hz and 800 Hz groups (Figure 2(b)). However, on day 14, AFVS at 800 Hz stimulated the highest level of mineralization (by 21.3% compared with the static group). Mineralized areas in cells treated with AFVS at 30 Hz and 400 Hz declined by 11.7% and 25.8%, respectively, compared with the static group (Figure 2(c)).

**Figure 3:**
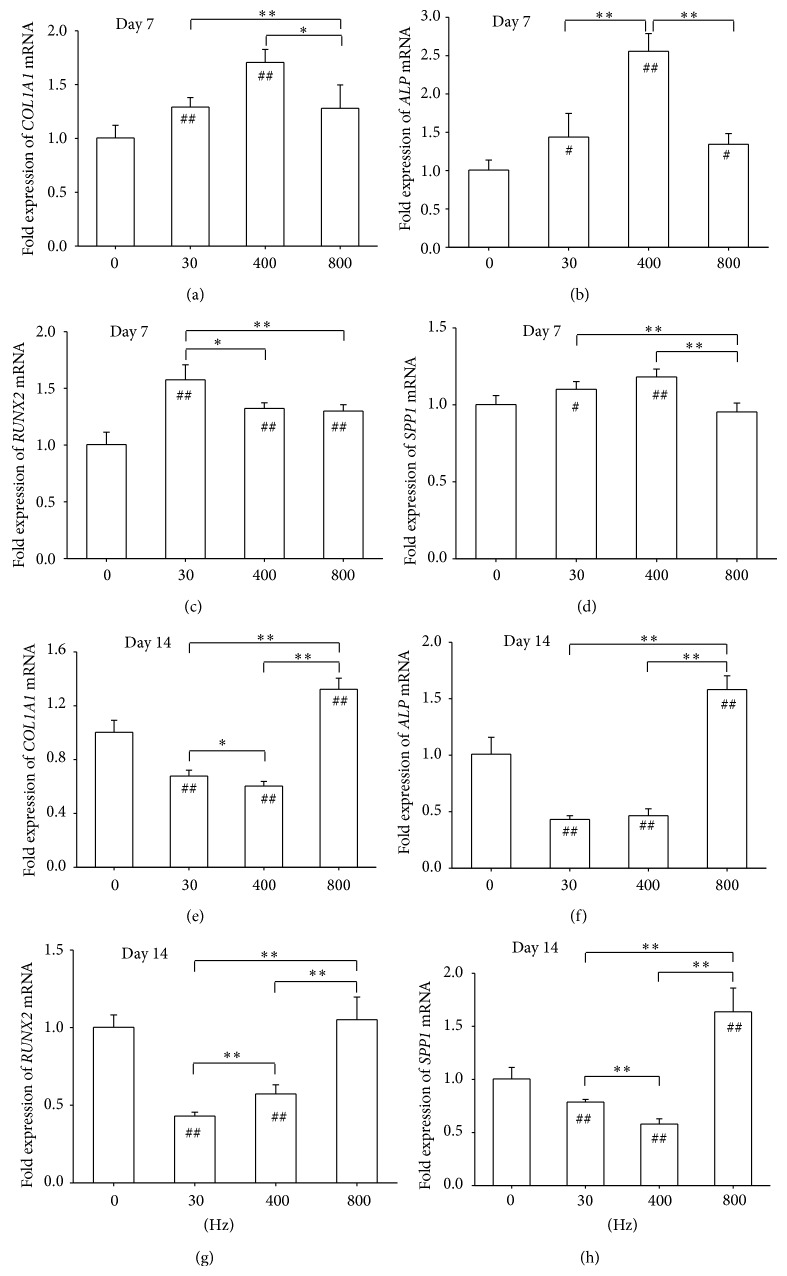
Acoustic-frequency vibratory stimulation (AFVS) modulates expression of mRNA encoding osteogenesis-specific markers in human bone marrow-derived mesenchymal stem cells (BM-MSCs) at the time points of day 7 ((a), (b), (c), and (d)) and day 14 ((e), (f), (g), and (h)). The mRNA levels of* COL1A1* ((a), (e)),* ALP* ((b), (f)),* RUNX2* ((c), (g)), and* SPP1* ((d), (h)) were measured by real-time RT-PCR. Values are mean ± standard error of four independent experiments (*n* = 4). ^*^ *P* < 0.05, ^**^ *P* < 0.01 in the indicated groups from unpaired *t*-test. ^#^ *P* < 0.05, ^##^ *P* < 0.01 compared with the 0 Hz control group from unpaired *t*-test.

To evaluate the extent to which AFVS affected osteogenic differentiation, transcript levels of osteoblast-specific genes were examined. On day 7, AFVS at 30 Hz and 400 Hz increased expression of* COL1A1* by 29.1% and 70.6%, respectively, compared with the static group (Figure 3(a)). Expression of ALP was elevated 1.56-fold higher by AFVS at 400 Hz relative to the corresponding untreated cells (Figure 3(b)). The transcription factor RUNX2 plays a critical role in MSC osteogenic differentiation, and AFVS at 30 Hz, 400 Hz, and 800 Hz significantly upregulated the level by 57.6%, 32.3%, and 30.0%, respectively, in contrast to the static group (Figure 3(c)). Similarly, AFVS at 30 Hz and 400 Hz increased the mRNA level of* SPP1* (Figure 3(d)).

On day 14, AFVS at 800 Hz significantly increased expression of* COL1A1* by 32.2% compared with the static group whereas AFVS at 30 Hz and 400 Hz downregulated the expression by 32.2% and 39.6%, respectively (Figure 3(e)). Regarding expression of* ALP*,* RUNX2*, and* SPP1*, AFVS at 800 Hz elevated the level of transcription by 58.1%, 5.1% (*P* = 0.582), and 63.7%, respectively, relative to corresponding expression of 0 Hz cells. However, AFVS at 30 Hz and 400 Hz showed inhibitory effects on the expression of these three genes (Figures 3(f), 3(g), and 3(h)).

### 3.3. AFVS at 800 Hz Suppresses Adipogenic Differentiation in BM-MSCs on Day 21

**Figure 4:**
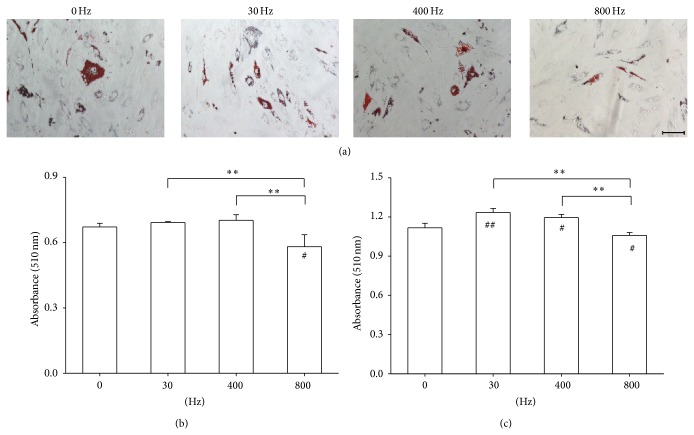
Effects of acoustic-frequency vibratory stimulation (AFVS) on lipid accumulation in adipocyte-like cells. Human bone marrow-derived mesenchymal stem cells (BM-MSCs) were incubated in adipogenic differentiation medium for 21 days and stimulated with AFVS at the frequencies of 30 Hz, 400 Hz, and 800 Hz. Cells in static culture condition (0 Hz) served as a control. (a) At the time point of day 21, lipid accumulation was assessed by Oil Red O staining. Scale bar = 200 *μ*m. The lipid-bound stain at the time point of day 14 (b) and 21 (c), respectively, was dissolved in isopropanol and quantified via a spectrophotometer. Values are mean ± standard error of four independent experiments (*n* = 4). ^*^ *P* < 0.05, ^**^ *P* < 0.01 in the indicated groups from unpaired *t*-test. ^#^ *P* < 0.05, ^##^ *P* < 0.01 compared with the 0 Hz control group from unpaired *t*-test.

Next, we investigated the effect of AFVS at different frequencies on adipogenesis of BM-MSCs. At the day 14 and day 21 time points, lipid accumulation was qualitatively and quantitatively determined by the Oil Red O stain. On day 21, adipocyte-like cells treated by AFVS at 800 Hz showed a smaller and lighter stain area compared with the 0, 30, and 400 Hz groups (Figure 4(a)). On both day 14 and day 21, AFVS at 800 Hz induced less lipid accumulation in differentiated BM-MSCs in contrast to the static group. However, AFVS at 400 Hz and AFVS at 30 Hz on day 21 increased lipid accumulation by 7.0% and 10.5%, respectively, relative to corresponding populations of static cells (Figures 4(b) and 4(c)).

**Figure 5:**
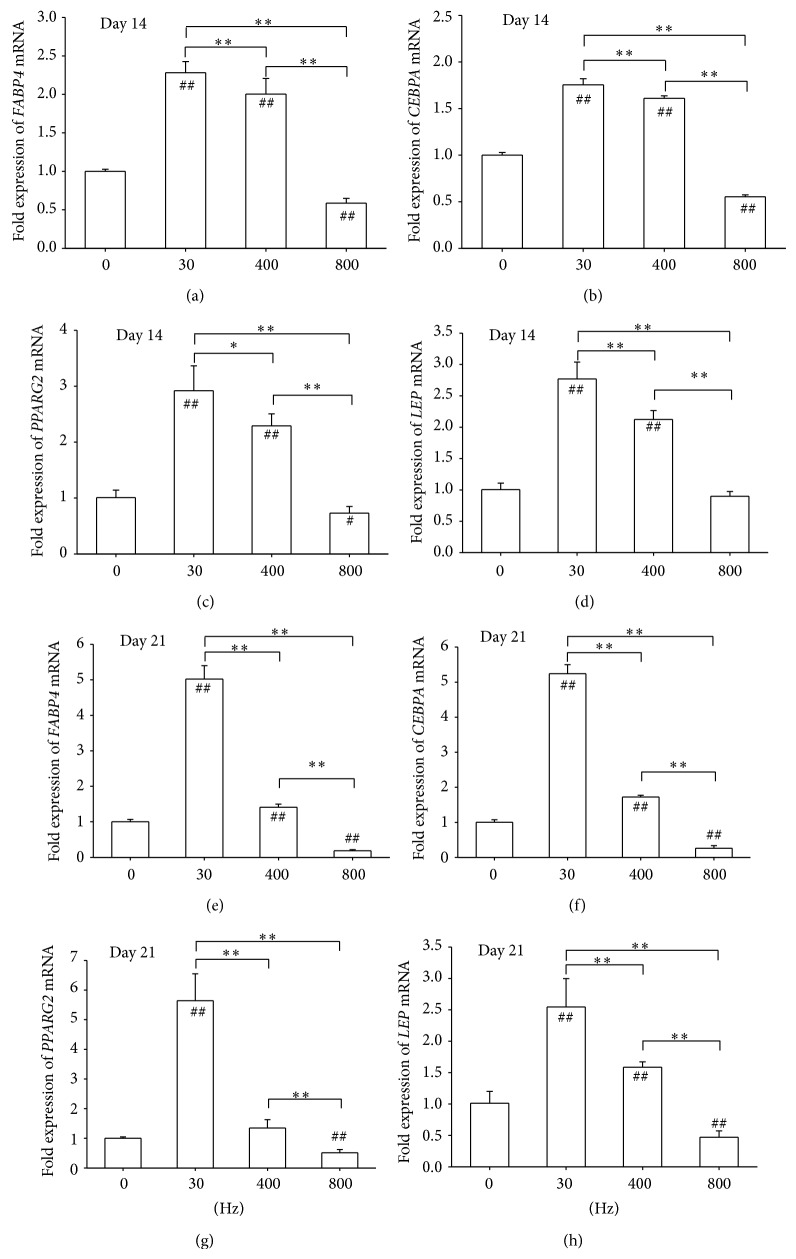
Acoustic-frequency vibratory stimulation (AFVS) modulates expression of mRNA encoding adipogenesis-specific markers in human bone marrow-derived mesenchymal stem cells (BM-MSCs) at the time points of day 14 ((a), (b), (c), and (d)) and day 21 ((e), (f), (g), and (h)), respectively. The mRNA levels of* FABP4* ((a), (e)),* CEBPA* ((b), (f)),* PPARG2* ((c), (g)), and* LEP* ((d), (h)) were measured by real-time RT-PCR. Values are mean ± standard error of four independent experiments (*n* = 4). ^*^ *P* < 0.05, ^**^ *P* < 0.01 in the indicated groups from unpaired *t*-test. ^#^ *P* < 0.05, ^##^ *P* < 0.01 compared with the 0 Hz control group from unpaired *t*-test.

Expression of adipocyte-specific marker genes, such as* FABP4*,* CEBPA*,* PPARG2*, and* LEP*, was determined by real-time RT-PCR. On day 14, AFVS at 30 and 400 Hz promoted levels of* FABP4 *by 1.3-fold and 1.0-fold, respectively, compared with the static control group, whereas AFVS at 800 Hz decreased the expression by 41.3% (Figure 5(a)). Similarly, AFVS at 30 and 400 Hz upregulated levels of* CEBPA*,* PPARG2*, and* LEP* while AFVS at 800 Hz suppressed expression (Figures 5(b), 5(c), and 5(d)). On day 21, the AFVS 30 Hz group generated the highest level of adipogenic markers by 4.0-fold for* FABP4*, 4.2-fold for* CEBPA*, 4.6-fold for* PPARG2*, and 1.5-fold for* LEP*, in contrast to the static group (Figures 5(e), 5(f), 5(g), and 5(h)). In agreement with Oil Red O stain results, AFVS at 800 Hz significantly downregulated levels of adipogenic genes by 81.6% for* FABP4*, 74.0% for* CEBPA*, 49.1% for* PPARG2*, and 54.1% for* LEP*, relative to corresponding populations of the control cells (Figures 5(e), 5(f), 5(g), and 5(h)).

## 4. Discussion

To our knowledge, this is the first study to report that mechanical vibration at acoustic frequencies can modulate lineage-specific differentiation in BM-MSCs. More specifically, osteogenesis is promoted but adipogenesis is inhibited by AFVS at the frequency of 800 Hz. Mechanical force vibration has been shown to promote bone formation and treat bone loss arising from disability or osteoporotic conditions under a vibration (<100 Hz) to the human whole body in preclinical and clinical trials [19]. However, the whole body vibration experienced significant energy absorption from the soft tissues, leading to a declination of the vibration efficiency in bone. Furthermore, most internal organs have their low frequency of resonance, so inappropriate whole body vibration may cause much more serious damage to the patients [20]. In contrast, the higher frequency is more suitable for local vibration. Sonic waves can be emitted locally to the area where the treatments are required, for example, the proximal femur or spine. While acoustic frequencies cover a wide range of 20–20,000 Hz, we found that three frequencies at 30, 400, and 800 Hz resulted in the same vibration amplitude of the cell culture plates. Therefore, we selectively used AFVS at these three frequencies as the first step to explore how BM-MSCs respond to vibration loading in this study. We found that AFVS did not damage cell viability but also vibration at 800 Hz showed an improved effect on cell proliferation.

BM-MSCs hold the capacity to differentiate into multiple cell types and thus they are considered ideal therapeutic cell sources for regenerative medicine and tissue engineering. We chose to use BM-MSCs because, as the progenitor cells of osteoblasts and adipocytes, the balance between osteogenic and adipogenic differentiation is closely related to the stability of bone remodeling. Once BM-MSCs are driven to differentiate toward one cell type, another cell fate is simultaneously inhibited [21]. In the case of osteoporosis, BM-MSCs gradually lose the ability of osteogenic differentiation but its ability of adipogenic differentiation increases. The distribution of balance has been considered as one of the reasons of osteoporotic generation [22].

The period of osteogenic differentiation was planned for a continuous 21 days [23] in accordance with adipogenesis; however, we found that osteoblast-like cells dissociated from cell plates after 2 weeks, so BM-MSCs were induced toward osteoblasts for only 14 days [18]. The cause of this phenomenon was possibly due to ECM accumulation and vibratory stimulation. As osteogenesis proceeded, coincidentally, the adhesive strength between the ECM and cell culture surface became weaker. Meanwhile, mechanical stimulation may accelerate the detachment of differentiated cells and ECM from culture plates. Thus, we will modify tissue culture polystyrene plates to increase the adhesive force [24] and analyze the long-term effect of AFVS on MSC osteogenesis.

The staining for mineralized ECM was barely visible at the time point of day 7 (data not shown), but the quantification of dye-bound mineral layers showed that AFVS at the frequencies of 30 and 400 Hz promoted matrix mineralization. Several previous studies demonstrated that low-magnitude vibrations at the frequency of 30 Hz facilitate the osteogenic process in preosteoblast cell lines [25] and MSCs [26]. Dumas et al. demonstrated that mechanical vibration at the frequency of 400 Hz enhanced osteogenic differentiation of C3H10T1/2 MSCs both at mRNA and protein levels [27], which is consistent with our results. Nevertheless, our study indicated that 30 Hz could indeed promote osteogenesis of BM-MSCs at early stage but suppress osteogenesis at late stage. Interestingly, our data also showed that 800 Hz had no apparent effect on osteogenesis on day 7 but improved osteoblast maturation on day 14. The osteogenic process of MSCs is usually divided into three distinct stages: osteoblast-like cell formation, matrix production, and matrix mineralization [28]. Thus, we speculate that mechanosensitive receptors on BM-MSC cell surfaces undergo a drastic change during osteogenic differentiation and therefore respond to different frequencies at the different stages. Future studies will be conducted to determine what changes in the mechanosensitive receptors occur during osteogenesis as well as the underlying mechanism for sensing mechanical stimuli.

Osteogenic and adipogenic differentiation of BM-MSCs are opposite processes, and our results revealed that 30 Hz stimulation favored cell adipogenic differentiation, in agreement with previous studies [29]. More importantly, we found that 800 Hz mechanical vibration suppressed the commitment of BM-MSCs to adipogenic lineage, as demonstrated by decreased transcripts of* PPARG/PPAR-*γ*2*, which is a master regulator of adipogenesis and osteogenesis. It has been demonstrated that PPAR-*γ*2 binds to the C/EBP*α* promoter to activate its expression and accelerates the process of adipogenesis [30], while silencing PPAR-*γ*2 through small interfering RNAs leads to retardation of adipogenesis but improvement of osteogenesis [31]. Meanwhile, the 800 Hz vibration induced commitment of BM-MSCs to osteogenic lineage accompanied by increased RUNX2 expression, which acted as an essential activator to induce osteogenic differentiation. The ERK1/2 signaling pathway may be involved in AFVS-induced BM-MSC osteogenesis. Zhou et al. showed that high-frequency vibration induced sustained phosphorylation of ERK1/2 during the process of osteogenesis and blockage of ERK1/2 transduction resulted in decreased alkaline phosphatase activity [32]. Another potential mechanism of mechanotransduction may relate to Wnt activation, especially Wnt10b, which was simultaneously up-regulated in MC3T3-E1 preosteoblast cells with exposure to vibrations [33]. However, the cellular signaling pathways regulating vibration-induced cell differentiation remain unclear and further investigation may elucidate underlying molecular mechanisms that determine cell fate by biasing the lineage commitment of BM-MSCs.

## 5. Conclusions

Our study provided a first glimpse at the influence of AFVS on commitment of BM-MSCs to osteogenesis and adipogenesis. BM-MSCs showed a frequency-dependent response to mechanical vibration. Acoustic vibration at 800 Hz but not 30 Hz nor 400 Hz was the most favorable for osteogenic differentiation and simultaneously inhibited adipogenesis. Further studies are necessary to verify the long-term viability and commitment to lineage-specific differentiation of BM-MSCs, with the purpose of determining whether acoustic vibration could potentially become a novel means to treat osteoporosis.

## Figures and Tables

**Table 1:** Primers used for real-time RT-PCR.

Gene	Primer sequence (5′-3′)	GeneBank accession
The internal standard gene		
*GAPDH *	F: AGAAAAACCTGCCAAATATGATGAC	NM_002046
	R: TGGGTGTCGCTGTTGAAGTC	

Osteogenic marker genes		
*COL1A1 *	F: CAGCCGCTTCACCTACAGC	NM_000088.3
	R: TTTTGTATTCAATCACTGTCTTGCC	
*ALP *	F: AGCACTCCCACTTCATCTGGAA	NM_000478.3
	R: GAGACCCAATAGGTAGTCCACATTG	
*SPP1/OPN *	F: GCGAGGAGTTGAATGGTG	NM_013227.2
	R: CTTGTGGCTGTGGGTTTC	
*RUNX2 *	F: AGAAGGCACAGACAGAAGCTTGA	NM_001015051.3
	R: AGGAATGCGCCCTAAATCACT	

Adipogenic marker genes		
*FABP4 *	F: AACCTTAGATGGGGGTGTCCTG	NM_001442.2
	R: TCGTGGAAGTGACGCCTTTC	
*CEBPA *	F: CGGTGGACAAGAACAGCAAC	NM_004364
	R: CGGAATCTCCTAGTCCTGGC	
*LEP/Leptin *	F: ATGACACCAAAACCCTCATCAA	NM_000230.2
	R: GAAGTCCAAACCGGTGACTTT	
*PPARG2 *	F: CCTATTGACCCAGAAAGCGATT	NM_015869.4
	R: CATTACGGAGAGATCCACGGA	
